# Promoter Hypermethylation of Estrogen Receptor Alpha Gene Is Correlated to Estrogen Receptor Negativity in Iranian Patients with Sporadic Breast Cancer

**Published:** 2012-08-31

**Authors:** Pantea Izadi, Mehrdad Noruzinia, Morteza Karimipoor, Mohammad Hamid Karbassian, Mohammad Taghi Akbari

**Affiliations:** 1. Department of Medical Genetics, School of Medical Sciences, Tarbiat Modares University, Tehran, Iran; 2. Department of Molecular Medicine, Biotechnology Research Center, Pasteur Institute of Iran, Tehran, Iran; 3. Day Hospital, Tehran, Iran

**Keywords:** Estrogen Receptor, Methylation, Breast Cancer

## Abstract

**Objective:**

Breast Cancer is the most common cancer in Iranian women. Breast tumors are classified based on the estrogen receptor alpha (ERα) expression status into ER negative and ER positive tumors. ER negative tumors tend to have worse prognosis and less likely to respond to endocrine therapy. Aberrant methylation of gene promoter is one of the mechanisms for gene silencing in breast tumors. Because of its reversible nature, promoter methylation is a good target for new therapeutic strategies. We aimed to evaluate the frequency of this epigenetic event in ERα gene and its association to clinicopathological features in Iranian breast cancer patients.

**Materials and Methods:**

In this case control study the patient series consisted of 100 sporadic primary breast cancer cases (51 ER negative and 49 ER positive tumors). None of the participants had chemo or radiotherapy before surgery. In breast tumors ERα promoter methylation were assessed with methylation specific polymerase chain reaction (MSP). Data was collected on clinicopathological features of the patients. Correlation between ERα methylation and clinicopathological characteristics of the patients was investigated by Pearson Chi-Square and Fisher’s exact test.

**Results:**

ERα methylation was detected in 98% of ER negative and 65% of ER positive breast tumors. A strong correlation was found between ERα methylation and ER negativity in tumors (p<0.0001). Also, ERα methylation has associated to progesterone receptor negativity (p<0.008) and double receptor negative status (p<0.0001) in breast tumors.

**Conclusion:**

ERα methylation occurs with high frequency in the breast tumors of Iranian breast cancer patients and may play a considerable role in pathogenesis of ERα negative tumors as a poor prognosis and more aggressive category. The reversible nature of DNA methylation may provide new therapeutic possibilities in ER negative breast tumors.

## Introduction

Breast Cancer, as the most common cancer in Iranian women, affects women at least one decade younger than their counterparts in developed countries (1). The highest frequency of this malignancy has been observed in the 40-49 years old age group (2).

It is commonly accepted that estrogen and its receptor have an important role in the pathogenesis of breast malignancies. Estrogen receptor alpha (ERα) expression classifies breast tumors into ER negative and ER positive cancers. About 40% of breast tumors are ER negative (3). ER negative tumors have poor prognosis in comparison to ER positive tumors. This type of tumor is more prevalent in younger patients and is not responsive to endocrine therapies.

Considering to the challenging nature of ERα negative tumors treatment and their innate poor prognosis, clarification of the molecular mechanisms that control expression of ERα is essential. This knowledge may enable us to modify the situation as such to restore sensitivity to endocrine therapies which provide us new opportunities for therapeutic options for ERα negative breast tumors (4).

Despite of many studies on the mechanisms of negativity of ER in breast tumors, many details still need to be clarified (5, 6). The loss of ERα expression in breast cancer may result from different underlying causes, such as structural changes within the gene or transcriptional silencing (7). Abnormalities, such as point mutations, deletions, loss of heterozygosity or polymorphisms within the gene have not shown to be frequent enough to explain ER negativity phenotype (7, 8). In breast tumors such as other types of cancer, epigenetic alterations are common and related to gene expression modification (9). It has been shown that tumor suppressor genes promoter methylation gives growth advantage to malignant cells (10). Because of the potential reversible nature of epigenetic gene silencing, epigenetic mechanisms have been under intense investigations in the recent years (5, 7, 11, 12). Regarding to evidences which have been resulted from several *in vitro* and *in vivo* studies, it has been shown that the inhibitors of DNA methyltransferase and histone deacetylase enzymes can reactivate ERα gene transcription in ERα negative cells. These epigenetic therapies could restore response to endocrine therapy in non responsive ER negative cells (13, 14). These promising landscapes have encouraged researchers to focus on the relationship between ERα negative phenotype and ERα promoter methylation. However, heterogeneity of the cellular population in breast tumors,, differences in methodological approaches, and variation of the studied populations (e.g. environmental exposures and ethnicity) have resulted in various reported frequencies for ERα methylation and its relevance to clinicopathological parameters (7, 11, 12).

Improved understanding of the mechanisms involved in ER negative breast tumors may permit improved therapeutic choices in this poor prognosis and more aggressive type of breast cancer. Thus, in the present study we aimed to evaluate the prevalence of epigenetic silencing of ERα via promoter methylation in Iranian patients with breast cancer and its association to ER negativity of tumors and other clinicopathological characteristics.

## Materials and Methods

### Patients

In this case control study the studied population consisted of 100 sporadic primary breast cancer patients referred to the Cancer Institute of Tehran University of Medical Sciences. This study was approved by the local Ethical Committee at Tarbiat Modares University. Written consent was taken from all patients enrolled in the study. Primary breast tumor tissues were provided by the Iran National Tumor Bank. Our inclusion criteria were sporadic and primary breast cancer. Patients who had recurrent breast tumor, had chemo or radiotherapy before surgery, or showed breast and/or ovarian cancers in first or second degree relatives were excluded from the study. Tumor samples were obtained through a surgical resection, then an expert pathologist performed rapid macro dissection of samples and transferred tumor tissues to liquid nitrogen reservoir, immediately.

### Clinical and pathological data collection

The main clinicopathological features of the study population were collected. These features included age at diagnosis, menopausal status, tumor size, histological type, grade, lymph node involvement, stage and immunohistochemistry (IHC) panel (ER, PR and her2). To overcome inter laboratory variations, a pathologist decided the histological type, grade and immunohistochemistry evaluations, then another expert rechecked and approved them. ER and PR were considered negative when nuclear staining of tumor cells was less than 1%. In tumors which complete and intense membrane staining were determined in the more than 10% of tumor cells, Her2 over expression was considered positive.

### DNA extraction and bisulphite modification

Genomic DNA was extracted from the breast tumors stored in liquid nitrogen tank. DNA was obtained by High pure PCR template preparation kit (Roche, Germany). Quantity and quality of the extracted DNA were evaluated by a spectrophotometer (Nano drop 2000, Thermo Scientific, USA). For each sample, 1 µg of genomic DNA was used for sodium bisulphite modification as previously described (15).

### Methylation specific PCR

Two regions of ERα promoter in CpG Island were studied by methylation-specific PCR (MSP). These regions were selected based on the previous studies. ER3 and ER5 regions were included in the 5'LUTR of proximal promoter of ERα gene (7, 12, 16). Two separate MSP reactions were carried out for each region, like one with primers specified for the methylated sequence (M) and the other with primers for the unmethylated sequence (U). Primer pairs and PCR condition were previously described (7). MSP reaction was optimized in our lab with 1'~PCR buffer, 6.7 mM MgCl2, and 1.25 mM dNTP per 25 ƒÊl reaction volumes. After initial denaturation, 2.5 units Taq DNA polymerase (Cinaclone, Iran) was added to each reaction (manual hot start). Positive MSP control for methylated specific primers was DNA extracted from MDA-MB-231 cell line. It was shown in the previous works that this DNA was totally methylated in 5'LUTR of ERα (7). Negative MSP control for methylated specific primers was the extracted DNA from MCF-7 cell line which was shown in the previous works to be totally unmethylated in our target regions in 5'LUTR of ERα (7). No DNA control was used in both methylated and unmethylated MSP reactions as the blank to show absence of template contamination. Untreated DNA was used as the control of specificity of reactions. MSP amplification products were electrophoresed on a 2% agarose gel with 1XTBE buffer, stained with ethidium bromide and visualized under a UV light. A tumor was considered methylated if a band was visualized in the MSP reaction with methylated primers.

### Statistical analysis

Correlation between ERα methylation and clinicopathological characteristics of the patients was investigated by Pearson Chi-Square and Fisher'fs exact test using SPSS version 13. The value of p<0.05 was considered significant and confidence intervals quoted were at the 95% level.

## Results

### Methylation frequency of ERα promoter in sporadic breast carcinomas

The methylation status of ERα promoter in ER3 and ER5 regions was determined in the study population using the MSP method. In analyzed 100 tumors, 71% were methylated in ER3 region and 56% in ER5 region. Tumors were classified as methylated if their bisulfate treated DNA had amplification with either one or both methylated primers. According to this definition, overall ERα methylation was detected in 88 tumors (88%). Breast tumors methylated in ER3 region were 1.3 times more than ER5 region.

### Correlation of ERα promoter methylation and ER negativity by IHC

To determine if there is a correlation between ER methylation and loss of ER protein expression in tumors (based on IHC results), we compared methylation frequency between ER negative and ER positive tumors. According to IHC results, 51% of samples were negative for ER expression. A strong correlation was found between ERα methylation in ER3 region and ER negativity in tumors. It was observed that 90% of ER negative versus 51% of ER positive cases were methylated in the mentioned region (p<0.0001). Frequency of methylation in ER5 region was lower than ER3 region in both ER negative (69%) and ER positive (43%) tumors. Overall, ERα methylation in both regions was significantly higher in ER negative tumors than ER positive cases. As we observed, 61% of ER negative tumors versus 28% of ER positive tumors were methylated in both regions synchronously (p<0.0001).

### ERα promoter methylation and correlation with clinicopathologic features

The clinicopathological characteristics of the 100 primary breast tumors are described in the table 1. There were significant correlations between estrogen receptor negativity in tumors and methylation in ER3 and ER5 regions in ER promoter. No correlation was found between the followings: ERα methylation status, age, menopausal status, tumor size, grade, nodal involvement, TNM stage and Her2 status. However, there was a significant correlation between methylation in ER3 region and negative status of progesterone receptor by IHC in tumors (p<0.008). In addition, presence of double receptor negativity by IHC in tumors (ER negative/PR negative status) showed a significant correlation with ER3 methylation (p<0.0001).

**Table 1:** Correlation between ERα methylation in ER3 and ER5 regions and clinicopathologic features of patients


Features	ER3 status		P value	ER5 status		P value
	M	U		M	U	

Age at diagnosis (Year)						
≤50 (n=56)	41	15	0.4	30	26	0.4
>50 (n=44)	30	14		26	18	
Menopause status						
Pre-menopause (n=50)	36	14	0.5	27	23	0.4
Post-menopause (n=50)	35	15		29	21	
Tumor size (cm)						
≤2.0 cm (n=16)	13	3	0.6	10	6	0.8
2.1-4.9 cm (n=57)	39	18		31	26	
≥5 cm (n=27)	19	8		15	12	
Grade						
I (n=25)	16	9	0.6	11	14	0.3
II (n=42)	31	11		24	18	
III (n=33)	24	9		21	12	
Nodal status						
Node-negative (n=38)	26	9	0.7	20	15	0.7
1-3 node (n=25)	20	8		16	12	
4-9 node (n=20)	14	6		10	10	
≥10 node (n=19)	9	6		8	7	
Stage						
I (n=11)	8	3	0.9	6	5	0.1
II (n=46)	34	12		30	16	
III (n=40)	27	13		18	22	
IV (n=3)	2	1		2	1	
Tumor type						
Ductal (n=95)	67	28	0.5	54	41	0.4
Non ductal (n=5)	4	1		2	3	
Estrogen receptor status						
Negative (n=51)	46	5	0.0001	35	16	0.008
Positive (n=49)	25	4		21	28	
Progesterone Receptor status						
Negative (n=68)	54	14	0.008	39	29	0.4
Positive (n=32)	17	15		17	15	
ER/PR status						
ER-/PR- (n=51)	46	5	0.0001	35	16	0.008
ER+/PR- (n=17)	8	9		4	13	
ER+/PR+ (n=31)	17	14		17	14	
ER-/PR+ (n=1)	0	1		0	1	
Her2 status						
Her2 negative (n=60)	42	18	0.5	35	25	0.3
Her2 positive (n=40)	29	11		21	19	


M; Means presence of methylated region in MSP. U; Means presence of unmethylated region in MSP.

## Discussion

Breast cancer affects Iranian patients at least one decade earlier than western patients (1, 2, 17, 18). Estrogen receptor alpha negative tumors are more prevalent in young patients with a worse prognosis than ER positive tumors (3). Different mechanisms, including: absence of specific transcription factor, presence of repressors, and epigenetic changes in CpG Island are potentially associated with ER negative phenotype (5, 7, 19). Previous studies have shown that epigenetic silencing of ERα is a major mechanism for ER negative phenotypes in breast cancer cells (7, 11) and the reversible nature of this mechanism may provide new therapeutic avenues for ER negative breast tumors (13, 20).

In the present study, methylation status of two important regions of the ERα 5'LCpG Island with respect to ER expression was investigated. Previous studies have shown that ER promoter methylation has association with lack of ER expression in breast tumors (7, 12, 21-23). On the other hand, in a recent population based on case-control study on 200 breast tumors, it has been suggested that the relationship between DNA methylation in ER promoter and ER protein expression is weak. So, these researchers have concluded that ER methylation is unlikely to represent a major mechanism of receptor silencing (24). Because of the reversible nature of DNA methylation, if there is a major mechanism for ER silencing in breast tumors, it will potentially provide new options for ER negative tumors treatment in the future. Thus, we aimed to evaluate this relationship in our study population and reveal the strong correlation between ERα negativity (based on IHC results) and ERα methylation in concordance with the previous works (7, 12, 21, 23, 25, 26). It seems that ER methylation can be an important mechanism for ER silencing at least in Iranian breast cancer patients.

Methylation frequency of ERα gene in the present study was higher in comparison to the previous investigations in the different study populations (Table 2).

**Table 2:** Comparison of overall ERα methylation in breast tumors in various studies


References	Study population ethnicity	Investigated region(s) in ERα CpG island	ER methylation in ER positive tumors	ER methylation in ER negative tumors

Zhao et al. (2008) (12)	Chinese	ER1-ER5	26/69 (37.7%)	57/69 (82.6%)
Mirza et al.(2007)(21)	Indian	ER4	6/15 (40%)	15/21 (71%)
Wei et al. (2008) (23)	Caucasian and African-American	ER1, ER3, ER4, ER5	13/59 (22%)	45/59 (76%)
Li et al. (2006) (22)	Australian	Not specified	108/134 (81%)	49/54 (91%)
Pirouzpanah et al.(2010)(26)	Iranian	ER3	17/62 (27%)	28/30 (93%)
Lapidus et al.(1998)(7)	American	ER1-ER6	12/21 (57%)	11/11 (100%)
Present study	Iranian	ER3, ER5	32/49 (65%)	50/51 (98%)


The differences between ERα methylation frequencies in various studies may be due to variation in ethnicity and carcinogenic exposures of populations studied. Also, technical issues in MSP conditions, such as: annealing; temperature; cycles number and selection of targets regions in CpG Islands may influence on final frequency. We have shown presence of ER methylation in a sizable fraction of ER positive cases. This observation maybe correlated to cellular heterogeneity in breast tumors. Classification of breast tumors based on their ER status into ER negative and ER positive groups may cause partial loss of information (27). ER positivity in breast tumors is a dynamic phenotype and over the natural course of cancer progression, Estrogen Receptor can be lost and many ER positive tumors become ER negative (6). Resistance to endocrine therapy in a significant fraction of ER positive breast tumors and recurrence of many ER positive tumors as ER negative ones emphasize again that this group is more heterogeneous than expected. Presence of ERα methylation in ER positive tumors is a manifestation of this heterogeneity and may contribute to endocrine therapy resistance or recurrence. More investigations needed to clarify the contribution of ERα methylation in endocrine therapy resistance and recurrence in ER positive breast tumors.

Investigation of clinicopathological association with ERα methylation showed that this phenomenon is not an age-related event in our breast cancer patients (Table 1). The relationship between age and ERα methylation in different studies was controversial. In one study on 193 Australian patients, ERα methylation was associated with younger patient (22). In other studies there was no correlation between ERα methylation and age in breast tumors (11, 12, 21, 23, 28).

In our study, we did not see any correlations between ERα methylation, tumor size and cancer stage in concordance to other investigations (12, 21-23). But in one study, the comparison of DCIS (Ductal Carcinoma In Situ), invasive ductal carcinoma and metastatic lesions showed an increasing trend in ERα methylation with malignant progression (25). The significant correlation between methylation in ER3 region and progesterone receptor status in tumors was not unexpected due to the fact that progesterone receptor expression is under ER control. This relationship was not independent of ER status of tumor because this association was observed only in ER negative/PR negative tumors (not in ER positive/PR negative cases). Also, we did not see any correlation between progesterone status and methylation in ER5 region of ER promoter. It is known that the impacts of methylation in various CpGs in a CpG Island are not equal and methylation in some regions is more critical in gene silencing. It may be due to less influence of methylation in the ER5 region on ER gene expression. Although, finding underlying mechanism of this observation needs more investigations.

## Conclusion

Our result showed that methylation of ERα is a prevalent epigenetic phenomenon in Iranian breast cancer patients. Since 98% of ER negative tumors had methylation in ER3, ER5 or both regions, it seems that ERα was a major target of methylation in our population studied. Therefore, the role of ERα methylation in the etiology of ER negative phenotype, which might be regarded as a common phenomenon in Iranian patients, should be investigated further. Higher prevalence of ER methylation in Iranian patients may be due to environmental exposures or carcinogenic lifestyles which must investigate in the future surveys. Although DNA methylation in promoter region of a gene has equal impact with a mutation, epigenetic modifications are potentially reversible, so they are good targets for new therapeutic strategies.
